# Lipid Control before CABG and Its Association with In-Hospital Mortality

**Published:** 2011-02-01

**Authors:** S K Hosseini, M Mehrpooya

**Affiliations:** 1 Lipid Control and CABG Mortality Center, Department of Cardiology, Tehran Heart Center, Tehran University of Medical Sciences, Tehran, Iran

**Keywords:** Dyslipidemia, CABG, Lipid control, In-hospital mortality

## Abstract

**Background:**

Controlling risk factors such as dyslipidemia in patients with coronary artery disease, including candidates for coronary artery bypass grafting (CABG), is of great importance and has serious effects on CABG morbidity and mortality. The aim of this study was to evaluate lipid serum levels, comprising TG, LDL, and HDL, before CABG and their relation with in-hospital outcome.

**Methods:**

The clinical profiles of 3,593 patients in the hospital cardiac surgery databank who underwent isolated CABG between April 2006 and April 2008 were reviewed. Three components of lipid profile, including TG, LDL, and HDL serum levels, were checked at the time of hospitalization in all the patients. Lipid control was evaluated according to the published guidelines.

**Results:**

The mean LDL, HDL, and TG serum levels were 103.4±48.5, 40.9±16, and 168±87 mg/dl, respectively. Additionally, 487 (13.6%) patients had entire TG, LDL, and HDL serum levels within the acceptable range and in 668 (18.6%) patients, all of these components were within the uncontrolled range. After adjustment for confounders, in-hospital mortality in patients with uncontrolled TG, LDL, and HDL was higher than patients with controlled TG, LDL, and HDL (p value=0.042, OR=1.399, 95% CI =1.012-1.934).

**Conclusion:**

The high prevalence of uncontrolled lipids in our patient population is alarming. Regular and frequent pre- and post-operative visits to monitor and modify patient risk factors, including dyslipidemia, seem necessary. An increase in statin dosage or adjunctive therapy with other lipid lowering agents may be helpful. Attempts to maintain all lipids within the controlled range may have beneficial effects on hospital outcome.

## Introduction

The control of the risk factors of coronary heart disease (CHD) as a secondary prevention, is still very unsatisfactory and inadequate.[1] Its role in CHD control, however, even in decreasing post-coronary artery bypass grafting (CABG) morbidity and mortality, is currently acceptable. Sufficient control of low density lipoprotein (LDL) serum level, less than 100 mg/dl, is one of the highly recommended secondary prevention plans in patients with CHD, especially in those for whom CABG is scheduled.[2][3][4] Statin should be prescribed for all patients before undergoing CABG with a view to achieve an LDL-C serum level of less than 100 mg/dl in patients undergoing elective CABG and less than 70 mg/dl in high-risk patients.[5][6] Preoperative statin therapy in patients undergoing CABG not only improves the post-operative myocardial perfusion of bypassed areas,[7] but also has several beneficial effects on the patency of saphenous vein conduit grafts by improving the endothelial function and inhibiting in the proliferation of smooth muscle cells that could decrease the speed of atherosclerosis progression in the vein graft conduits in CABG.[8][9] Statin has also beneficial effects on decreasing perioperative and long-term mortality and morbidity of redo revascularization in CABG patients.[5] Nonetheless, there are still reports of inadequate lipid control in large proportions of these patients.[10][11][12] The aim ofthis study was to evaluate pre-CABG lipid control and its association with in-hospital outcome.

## Materials and Methods

From April 2006 to April 2008, 3,593 patients who underwent CABG were enrolled. All medical records of patients were derived from our hospital cardiac surgery databank.[13][14] The patients were referred by cardiologists of our center or other centers and clinics. The serum lipid levels were checked upon the patients’ hospitalization. All definitions of the variables were according to our hospital cardiac surgery data bank.[13][14] The inclusion criteria were coronary artery disease and candidacy for isolated CABG.

Uncontrolled lipids were defined as LDL of 100 mg/dl or more, triglyceride (TG) of 150 mg/dl or more, and high density lipoprotein (HDL) of 45 mg/dl or less in men and 55mg/dl or less in women. Statistical analysis was performed with SPSS-15 (SPSS –I ncm, Chicago, IL, USA) using the Chi-Square and t tests and analysis of variance. The univariate analysis of the continuous variables was carried out using Student’s T-test, and the categorical variables were compared using the Chi-Square or isher’s exact test. Multivariable logistic regression model for comparing mortality in the two groups of uncontrolled TG and LDL and HDL versus controlled TG and LDL and HDL in the presence of confounders was constructed. The association of uncontrolled lipid profile and mortality was expressed as odds ratio (OR) with 95 percent confidence interval. Differences were considered statistically significant when a p value was less than 0.05.

## Results

Among CABG patients, 2,695 (75%) cases were men and 898 (25%) were women with a mean age of 58.8±9.9 years. The demographic and characteristics of the patients are listed in Table 1. The status of surgery in these patients was as follows: 2817 (79.1%) elective, 741 (20.8%) urgent and 4 (0.1%) were emergent CABGs. In total, 2,554 (71.6%) patients had three-vessel coronary artery disease, 761 (21.3%) two-vessel disease, and 252 (7.1%) a single-vessel disease. Additionally, 273 (7.6%) patients had left main disease. The mean values of serum lipid levels were as follows: LDL of 103.16±39.1, HDL of 40.91±16.12, and TG of 168.8±87.09 mg/dl.

In 487 (13.6%) patients, the entire TG, LDL, and HDL serum levels were within the acceptable range; and in 668 (18.6%) patients, none of these components was controlled. In 1,032 (28.7%) patients LDL and HDL; in 389 (10.8%) patients LDL and TG; and in 474 (13.2%) patients HDL and TG serum levels were in the uncontrolled range (Table 1). Table 2 lists the cumulative frequency of the categorized LDL serum levels in the CABG patients. The total in-hospital mortality rate was 1.4 % (47 patients). The incidence of uncontrolled serum LDL, HDL, and TG in the patients who died was 48%, 87.7%, and 52.1%, respectively; compared respectively with 51.5%, 85.5%, and 50.8% of those who survived. No significant difference was noticed in lipid profile levels between the dead and surviving cases (p value>0.05). However, in-hospital mortality in the patients with controlled TG, LDL, and HDL was 0.6 % and in those with uncontrolled TG, LDL, and HDL was 2.4 %, which was significantly different (p value=0.019) (Table 3). After adjustment for confounding variables including age, body mass index (BMI), smoking, hypertension (HTN), diabetes mellitus (DM), angiographic ejection fraction (EF), history of renal failure (RF), history of peripheral vascular disease (PVD), history of pervious CABG, left main disease (LM) and three vessel disease (3VD) (Table 4); in hospital mortality rate remained higher among pat nts with uncontrolled TG, LDL, and HDL versus those with controlled TG, LDL, and HDL (Odds ratio=1.399, 95% CI =1.012- 1.934, p value=0.042). Regarding mortality between other groups, the difference was not significant (Table 3). Lipid lowering agents were prescribed in 3,126 (87.3%) patients post-operatively. These agents were not administered in 453 (12.7%) patients because of intolerance or other contraindications.

**Table 1:** Baseline patients' characteristics

**Characteristics**	**Men (No=2695) No (%)**	**Women (No=898) No (%)**	**Total No (%)**
Cigarette smoking^a^	1309 (48.6)	62 (6.9)	1371 (38.2)
Hypertension	1233 (45.9)	631 (70.3)	1864 (52)
Diabetes	778 (28.9)	325 (36.2)	1103 (30.7)
Hypercholesterolemia	1766 (65.7)	677 (75.8)	2443 (68.2)
Uncontrolled LDL (100 mg/dl≤)	1228 (46.6)	447 (51.6)	1675 (46.6)
Uncontrolled HDL (≤55 mg/dl in women ) (≤45 mg/dl in men)	2347 (87.1)	740 (82.4)	3087 (85.9)
Mean angiographic EF	48.48%	51.48%	49.26%
History of myocardial infarction	1232 (45.7)	340 (37.8)	1573 (43.8 )
History of renal failure	52 (1.9)	25 (2.7)	77 (2.1)
History of Cerebrovascular accident	91 (3.3)	61 (6.7)	153 (4.2)
History of peripheral vascular disease	69(2.5)	31 (3.4)	100 (2.7)
History of previous CABG	8 (0.3)	0	8 (0.2)

^a^ LDL, low density lipoprotein; HDL, High density lipoprotein; EF, ejection fraction; CABG, Coronary artery bypass grafting

**Table 2:** Commulative frequency of categorized LDL serum levels in CABG patients.

**LDL levels (mg/dl)**	**Frequency**	**Percentage^a^**	**Cumulative Percentage**
<70	685	19.6	19.6
70-99	1115	31.9	51.5
100-129	877	25.1	76.5
130-159	542	15.5	92
160-189	200	5.7	97.7
≥190	79	2.3	100
Total	3498^a^	100	

^a^ Valid percentage was used because about 2.5% was missing. LDL, low density lipoprotein

**Table 3:** Comparison between combined dyslipidemia and in-hospital mortality

**Groups^a^**	**Total cases**	**In hospital mortality (rate %)**
Controlled all Lipids	487	3 (0.61)
Uncontrolled one Lipid	543	8 (1.47)
Uncontrolled HDL and LDL	1032	9 (0.87)
Uncontrolled TG and LDL	389	5 (1.3)
Uncontrolled TG and HDL	474	6 (1.26)
Uncontrolled HDL, LDL and TG	668	16 (2.40)
All Cases	3593	47 (1.31)

^a^ The Comparison between “Controlled all Lipids” and “All Cases”: P>0.05(NS), The Comparison between “Con-trolled all Lipids” and “Uncontrolled HDL & LDL & TG”: P=0.019, The Comparison between “Uncontrolled HDL & LDL” and “Uncontrolled TG & LDL”: P>0.05(NS), The Comparison between “Uncontrolled HDL & LDL” and “Un-controlled TG & HDL”: P>0.05(NS), The Comparison between “Uncontrolled TG & LDL” and “Uncontrolled TG & HDL”: P>0.05(NS), The Comparison between “Uncontrolled HDL & LDL” and “All Cases”: P>0.05(NS), The Com-parison between “Uncontrolled TG & LDL” and “All Cases”: P>0.05(NS), The Comparison between “Uncontrolled TG & HDL” and “All Cases”: P>0.05(NS), NS, non-significant; P-value>0.05, LDL, low density lipoprotein; HDL, High density lipoprotein; TG, triglyceride

**Table 4:** Comparison of variables among survivors and dead patients

**Variable**	**Surviving (No=3546)**	**Dead (No=47)**	**P value**
Age	58.71±9.90	63.72± 9.10	0.001
Male gender	2663 (75.1%)	32 (68.1%)	0.270
BMI (Kg/m^2^)	27.42±4.06	25.73±4.48	0.005
Smoking a	1358 (39.4%)	12 (25.5%)	0.054
DM^a^	1198 (33.9%)	23 (48.9%)	0.031
HTN^a^	1833 (51.8%)	32 (68.1%)	0.026
(+)Family History^a^	1598 (45.6%)	20 (44.4%)	0.875
Angina	3272 (92.3%)	42 (89.4%)	0.409
Angiographic EF	49.45±9.45	46.05±11.16	0.019
Hx of CVA	150 (4.2%)	3 (6.4%)	0.452
Hx of PVD	95 (2.7%)	5 (10.6%)	0.009
Hx of CABG	6 (0.16%)	2 (4.25%)	0.004
Hx of MI	1552 (43.8%)	21 (44.7%)	0.903
LM disease	265 (7.47%)	8 (17.02%)	0.054
3VD^a^	2515(71.4%)	39 (86.7%)	0.024
Hx of renal failure	70 (1.97%)	5 (10.6%)	0.003
Triglyceride	173.3±32.8	170.5±27.7	0.842
LDL	98.16±35.2	101±28.1	0.658
HDL	42.6±9.1	40.12±6.5	0.958

^a^ There may be small percentage (less than 2%) missing rate in some variables. Data are presented as Mean±SD or No (%), CVA=cerebrovascular accident, PVD=peripheral vascular disease, Hx=history of, BMI=body mass in-dex, LDL=low density lipoprotein, HDL=high density lipoprotein, MI=myocardial infarction

## Discussion

Although the mean LDL serum level in our study was near the acceptable range, it was evident that LDL was not under control in about half of the study population. Also, in a relatively large proportion of this group of patients, HDL was less than the optimal level (<55 mg /dl in women and <45 mg/dl in men). LDL, HDL, and TG control was achieved in a small proportion of our patients. This fact may in part be due to the finding that about 21% of the patients had undergone urgent or emergent CABG and thus there may not have been enough time to reach the goal of control. This finding is in agreement with that of the Paraskevas and colleagues review study,[5] which stated that a large percentage of cardiothoracic surgical patients were suboptimally treated with statin. Another study reported that the prevalence of risk factors in CABG patients was very high; and despite a high level of medical treatment, risk factor management was very poor.[15] Another reason for this high percentage of uncontrolled lipid levels may be due to the insufficient effect of statin in some patients. As was stated in other studies,[16][17] increase in statin dosage or adding other lipid lowering agents as combination therapy such as ezetimibe or lipid binding resins could facilitate attaining favorable serum LDL levels in patients.

There is some debate about the role of lipid lowering therapy in the outcome of cardiovascular surgical procedures. In our study, the mean of TG, LDL, and HDL serum levels was not significantly different between the dead and surviving patients. Nevertheless, a statistically significant difference was observed in terms of in-hospital mortality in the patients with controlled TG, LDL, and HDL as opposed to the patients who had uncontrolled TG, LDL, and HDL. This is the original and main finding of the present study and chimes in with a recent systematic review showing that statin can be protective in patients undergoing cardiac and non-cardiac surgeries.[18] Another meta analysis of 13 controlled studies (including 3 randomized controlled trials) demonstrated that pre-operative statin therapy reduced post-operative mortality in patients with cardiac surgery.[19] On the other hand, Powell and colleagues stated that lipid lowering therapy before CABG was not associated with decreased inhospital mortality or morbidity, including cerebrovascular events, reoperation for bleeding, and postoperative atrial fibrillation.[20] Another study with findings that disagree with those in the present study looked into 1,308 matched patients after isolated CABG and concluded that pre-operative statin use did not reduce peri-operative mortality or morbid events.[21] It seems that more studies are required to further elucidate this controversial issues.

Discontinuation of pre-operative statin therapy following CABG is another important issue that could lead to adverse effects and mortality in comparison with patients whose statin treatment is continued following the procedure.[5][22] Lipid lowering agents were continued post-operatively in 87.3% of our study population.

There is still room for improvement in lipid control issue in CABG patients. Regular and frequent pre-operative and post-operative visits are recom- mended in order to lower serum LDL to suggested levels of less than 100 mg/dl and less than 70 mg/dl in high-risk patients by modern management of CHD patients. Maintaining TG, HDL, and LDL levels within the optimal range seems to have a positive bearing on CABG outcome. Statin therapy or even combination therapy based on patient conditions is suggested for achieving optimal lipid serum levels. Further studies are required and suggested for evaluating the optimal timing of elective surgery after commencing statin treatment to take full advantage of these agents.
